# Frequency Switchable Global RFID Tag Antennae with Metal Compatibility for Worldwide Vehicle Transportation

**DOI:** 10.3390/s23083854

**Published:** 2023-04-10

**Authors:** Krishna Mazumder, Anumoy Ghosh, Anagha Bhattacharya, Sarosh Ahmad, Adnan Ghaffar, Mousa Hussein

**Affiliations:** 1 Department of Electronics and Communication Engineering, National Institute of Technology, Aizwal 796012, Mizoram, India; 2 Department of Electrical and Electronics Engineering, National Institute of Technology, Aizwal 796012, Mizoram, India; 3 Department of Signal Theory and Communications, Universidad Carlos III de Madrid (UC3M), 28911 Madrid, Spain; 4 Department of Electrical and Electronic Engineering, Auckland University of Technology, Auckland 1010, New Zealand; 5 Department of Electrical Engineering, United Arab Emirates University, Al Ain P.O. Box 15551, United Arab Emirates

**Keywords:** RFID, frequency switching, PIN diode, metal mountable

## Abstract

This paper presents an effective way to design an RFID tag antenna to operate at three different frequencies by incorporating a switching technique. PIN diode has been used to switch the RF frequency because of its good efficiency and simplicity. The conventional dipole-based RFID tag has been improvised with added co-planar ground and PIN diode. The layout of the antenna is designed with a size of 0.083 $\lambda_{0}$ × 0.094 $\lambda_{0}$ at UHF (80–960) MHz, where $\lambda_{0}$ is the free-space wavelength corresponding to the mid-point of the targeted UHF range. The RFID microchip is connected to the modified ground and dipole structures. Bending and meandering techniques on the dipole length help to match the complex chip impedance with the dipole impedance. Additionally, it scales down the total structure of the antenna. Two PIN diodes are placed along the dipole length at appropriate distances with proper biasing. The ON-OFF switching states of the PIN diodes enable the RFID tag antenna to switch over the frequency ranges (840–845) MHz (India), 902–928 MHz (North America), and 950–955 MHz (Japan).

## 1. Introduction

Radiofrequency identification (RFID) has become popular among other auto-ID systems for decades due to the use of electromagnetic waves for auto-identification purposes. RFID has been dominating in different applications such as logistics, product management, and vehicle management [1]. RFID systems establish communication between two types of antennae: Tag antenna and reader antenna. A microchip is attached to the tag antenna which is used to store the information about the tag. Generally, the tag is placed on the object, and the reader antenna needs to detect and locate the tag [2]. The impedance of the microchip is complex, and therefore impedance matching between the antenna and integrated chip to design tag antennae is very challenging. RFID can communicate over a standard frequency band such as low frequency (125–134 kHz), high frequency (13.56 MHz), ultrahigh frequency (UHF) (840–960 MHz), and microwave frequency (2.45 or 5.8 GHz) [3]. UHF band is used in vehicle management all over the world which varies with countries such as (840–845) MHz (in India), (902–928) MHz (in North America), and (950–955) MHz (in Japan) [4,5].

In industrial provinces, for inter-country logistic transport, whether commercial or individual, multiband tag antennae would be useful for avoiding unnecessary changes of tags while entering a country from another as per the frequency allocation of RFID communication in the country of concern. Hence, multiband antennae at the UHF range are preferable. Proper impedance matching and high read range properties are desired in multiband RFID tag antennae [6]. Various dual band tag antennae have been reported in the literature, and these antennae employ different techniques such as F-shaped monopole RFID tag working at microwave frequency [7], conjoining PIFA and meandered microstrip patch antennae for both UHF and microwave frequencies [8], incorporating bent feedlines between concentric microstrip circular rings [9], conventional planar dipole tag with T-matching networks [5], and two distinct antennae for receiving and backscattering operations [10]. Additionally, the tag can be attached to any surface directly, and therefore metal compatibility is one of the major criteria for designing the tag antennae. To implement this criterion, there are numerous methods that have been investigated. Microstrip Patch [11] PIFA antennae [8] are preferable for ground-based structures. However, for low frequencies, the footprint of the above-mentioned antennae would be large. Hence dipole-based planar antenna is more advantageous than others. The radiation pattern of the dipole antennae is omnidirectional, which helps the tag to detect any angle of the visual sight. Above all, by applying the meandering technique on the dipole antennae, the total antenna dimension can be reduced. The association with metal plates degrades its performance in terms of radiation patterns [2]. Artificial Magnetic Conductor below the antenna structure is a well-known method to shield the antenna from the effect of the metal surface [12,13]. However, it increases the complexity volume of the total antenna structure. With the help of the shorting wall, the C-shaped radiating patch is connected to the ground and mounted on the metal plate (200 × 200 mm^2^) [14]. E-shaped radiators are connected to the ground surface with the help of inductive and capacitive stubs [15]. A pair of dipole patches on the top of the substrate and ring shape radiating elements on the backside antennae are placed on 20 cm × 20 cm metal plates [16]. Another pair of semicircular patches are also designed for metal insensitivity. It is designed on the double layer of the Roger substrate with a shorting stub [17]. The T-shaped folded patch is used on the double-layer substrate with a shorting stub [18]. A thin inductive plate is attached to the radiating patch with I-shaped slots, attached to the ground plate, and clamped at the center of the 250 × 250 mm^2^ metal surface [19]. Cavity-based UHF tag antennae with a T-matching network are designed for metal and non-metal surfaces [20].

The novelty of this work is to establish a synthesis of the RFID tag antenna with RF PIN diode to achieve switching operation within the UHF band so that the tag can be used in different countries according to the RFID communication frequency assigned in the respective countries thereby facilitating inter-country transportation of vehicles. There are numerous works on switching performances of an antenna using RF diodes [21,22]. Entire investigations of the above-mentioned designs are limited to 50 Ω port antennae that cannot be implemented in RFID tags since they use chips as feeding ports which have complex impedance. The proposed design circumvents this problem by using RF diodes for a complex impedance port antenna that enables the switching of the tag at three frequencies. Another important design improvisation over other conventional tag antennae is that in the proposed structure, the chip is incorporated with the antenna without any external impedance-matching circuits. The impedance matching is accomplished by meandering the radiating antenna and etching staircase slots on the ground. Finally, to mount the tag on metallic surfaces, a metal plate having the same dimension as the tag antenna is placed below the antenna at a certain distance that does not deteriorate the antenna’s performance. To the best of the knowledge of the authors, there has been no experimentally verified structure proposed in the literature for switchable tag antennae.

## 2. Antenna Geometry

**Figure 1:**
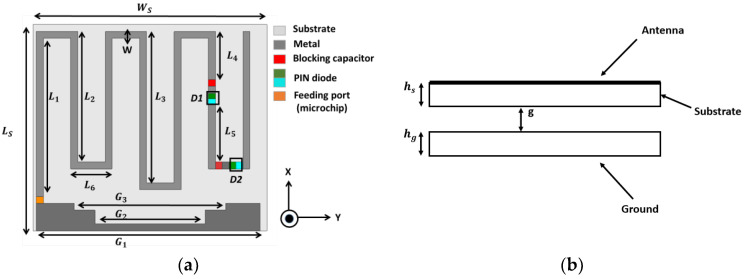
Geometry of frequency switchable RFID tag antenna (**a**) Top view (**b**) side view.

The proposed antenna structure is designed on an FR4 substrate of dielectric constant 4.4, height 1.6 mm, and loss tangent 0.02. The total layout dimension is 0.083 $\lambda_{0}$ × 0.094 $\lambda_{0}$. The antenna geometry is illustrated in Figure 1 with the relevant dimensions given in Table 1. In this work, the foremost concern is to switch the frequency over the UHF range. In order to impose the switching method on the tag antenna, the proposed design is incorporated with PIN diodes. Two PIN diodes (BAR6402V) [23] are used to interchange the frequencies among 840 MHz, 900 MHz, and 950 MHz to cover the Indian, North American, and Japanese frequency ranges to enable cross-country transport management. The structure of the antenna is dipole-type, with meandered arms for miniaturization. The dipole antenna has four sets of folded arms. PIN diodes are placed along the dipole pathway, as indicated in Figure 1. In order to establish proper biasing for the PIN diodes, the co-planar ground is added. A stair shape cut is given at the boundary of the ground plane to lower the frequency of the desired resonance. One blocking capacitor is connected in parallel with every switch such that it can suppress the DC signal. The microchip is connected between the dipole and the ground plane within an area of 0.5 mm × 0.5 mm, as shown in Figure 1a. The excitation is given by the chip from the input port to the tag antenna. The microchip of NXP UCODE8 m/G2iL [24] is used to store the required information and to operate at the broad international frequency range of 840–960 MHz.

The proposed structure is designed as a single-layer full wavelength dipole. A low-frequency wavelength is large. As a result, the antenna length is very long. Therefore, bending and folding the radiating path helps to reduce the overall layout dimension of the antenna structure. The input impedance of the tag antenna is complex in nature. On that account, their meandering shape improves their impedance without any external matching circuits. The effective wavelength for the required frequency is given as the following [25]:

$$\lambda =\frac{c}{\sqrt{\varepsilon_{r}}f}$$

Here,

*λ* = Wavelength.

*f* = Frequency.

*c* = Velocity of light.

$\varepsilon_{r}$ = Relative Dielectric constant.

Hence, the required length is L = *λ* + ∆l.

Here, ∆l is the extra length accounted for due to the fringing electric fields from the open ends of the dipole arms.

From Figure 1 of the proposed structure, the length of the full wavelength dipole is L = L_1_ + (L_2_ × 4) + (L_3_ × 2) + (L_6_ × 6) = *λ* + ∆l.

Since the designed chip antenna is intended to switch between three frequencies (840 MHz, 900 MHz, and 950 MHz), the chip impedance at each frequency will vary slightly. Hence, an average chip impedance of 22 − j224 Ω is considered for the design. Thus, for maximum power transfer, the dipole impedance should be a complex conjugate of the chip impedance, that is, 22 + j224 Ω. This indicates that the reactive impedance of the dipole should be inductive in nature and the necessary inductive impedance is achieved by tuning the parameters such as the width of the strips, height of the vertical arms, and space between vertical folded branches.

The next necessary step is to add a metallic plate beneath the antenna structure to make the antenna metal insensitive so that no degradation in antenna performance happens when it comes in close vicinity to any metal surface. The ground plane has dimensions of 30 × 34 mm. The distance between the antenna and the ground plane should be precise in order to keep the switching function and the radiation patterns of the tag antenna unperturbed. The optimum distance between the antenna structure and the ground plane is g = 5.4 mm.

### PIN Diode as Switch in the Proposed Antenna Design

**Figure 2:**
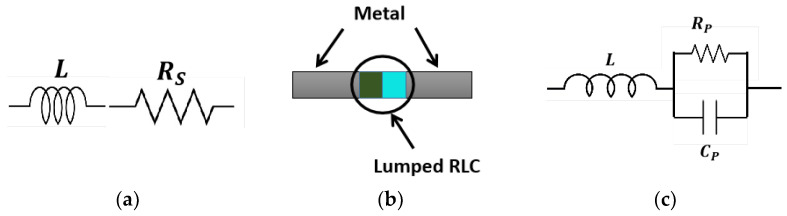
Equivalent circuit and layout of PIN diode: (**a**) ON condition; (**b**) Design format; (**c**) OFF condition.

The PIN diode is one of the favored ways to use in antennae for switching the resonant frequency. The reconfigurability of the resonant frequency of the antenna is accomplished by the pair of PIN diodes added to the thin strip of the monopole antenna. The model of the BAR64-02V PIN diode is considered here, which has an operating frequency range from 1 MHz to 6 GHz. Both the ON and OFF states of the PIN diode have package circuit parameters, such as L= 0.6 nH, R_S_ =2.1 Ω, R_P_ =3.9 KΩ, and C_P_ = 0.17 pF. The equivalent circuit for the ON and OFF states of the PIN diode is shown in Figure 2. When the diode is at the ON or forward-biased stage, it behaves as a low resistance component in series with inductance. On the other hand, in the OFF or reverse bias conditions, it behaves like a parallel combination of reverse resistances with capacitance. By applying the DC bias voltage of 3 V, the above-mentioned stages can be controlled. As two PIN diodes are used in four ways as ON/OFF combinations, the frequency can be switched to the operating frequency.

## 3. Results and Discussion

**Figure 3:**
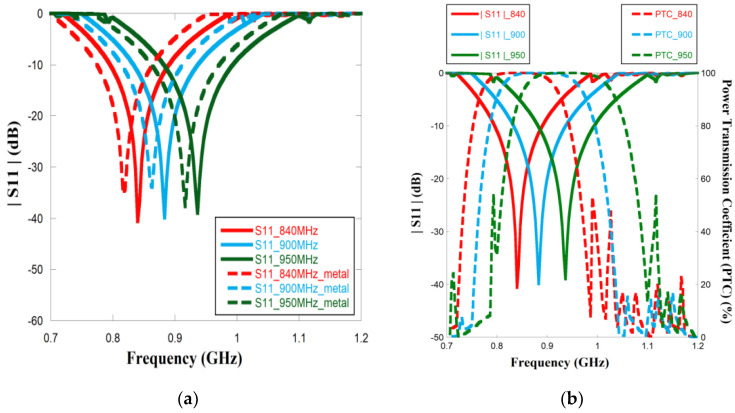
(**a**) Compared results of |*S*11| of metal vs without metal; (**b**) |*S*11| and PTC results for three frequencies.

Figure 3a exhibits the simulated |*S*11| characteristic of the proposed tag antenna with and without a metallic plate. The simulations are conducted for three switching conditions, that is, ON–ON, ON–OFF, and OFF–OFF. The detailed response is tabulated in Table 2. This table shows that by changing the bias conditions, the resonance of the tag antenna can be flexibly tuned within 840–960 MHz. The introduction of the metal has a very nominal effect on the |*S*11| characteristic.

The power transmission coefficient (Γ) is given by [26],

(1) $$\Gamma =(1-{\left|S_{11}\right|}^{2})$$

Using Equation (1), Γ is graphically depicted in Figure 3b. For the tag antennae with the metal plates. The figure indicates that at each switching condition, the power transmission is above 99% at the resonant bandwidth, thus indicating maximum power transfer from the chip to the antenna and vice versa due to good impedance matching. PIN diode status and corresponding resonant frequencies and impedance bandwidth are given in Table 2.

**Figure 4:**
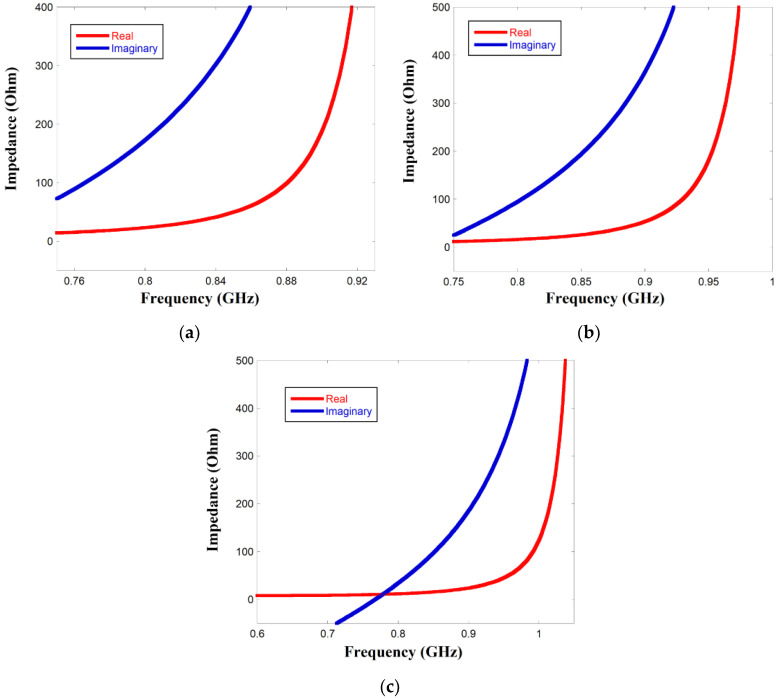
Input impedance (Real and Imaginary) of the proposed Tag antenna (**a**) 840 MHz (**b**) 900 MHz (**c**) 950 MHz.

The input impedance of the meandered monopole radiator with the metallic plate is illustrated in Figure 4 for all three switching frequencies. UCODEG/G2 iL microchip is considered and used in the proposed antenna. Table 3 compares the input impedance of the monopole antenna with the microchip at the switching frequencies. The table highlights that the antenna impedance is almost equal to the complex conjugate of the chip impedance at all the switching frequencies, thereby facilitating maximum power transfer from the chip to the antenna, which is confirmed by Figure 3b.

### 3.1. Parametric Study

**Figure 5:**
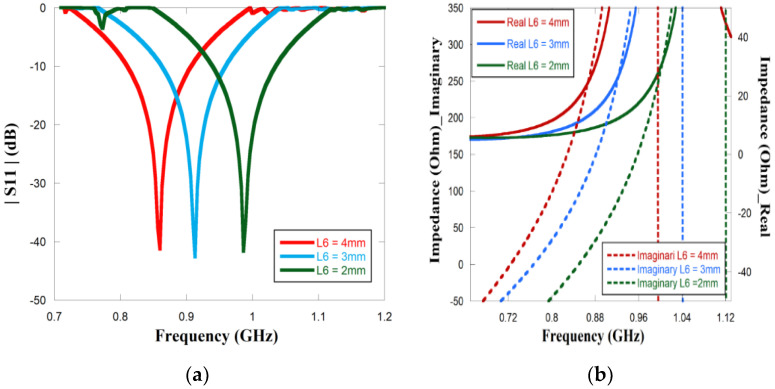
Parametric study of simulated (**a**) |*S*11| and (**b**) complex impedance for different L6.

The parametric effects of the proposed antenna have been investigated by simulation in ANSYS software. The meandering technique is considered to obtain a lower resonant frequency by means of the small-scale size of the structure. A long meandering path helps the antenna to match the complex conjugate impedance of the chip. The survey of changing the horizontal arms of the meandering layout exhibits the effect of resonant frequency along with the impedance of the antenna. Figure 4b and Figure 5a show the outcome of the resonant frequencies and complex impedance for different lengths of the arm. Since the length of the arm changes simultaneously, the wavelength of the antenna changes. It is clearly seen from Figure 5a that when the length L6 is alternated from 4 mm or 3 mm to 2 mm, resonant frequency also shifted from 849 MHz to 920 MHz and 989 MHz, respectively. On account of changing the horizontal meander arm, inductance and capacitance values were also affected as the vertical arms of the meandering path drew close to each other. Therefore, the complex impedance of the antenna also changed as the length of the arms decreased. Figure 5b depicts how the alternation of the impedance in particular reactance is increased as it moves to higher frequencies. The reactance of 218, 247, and 262 was accomplished at 849 MHz, 920 MHz, and 989 MHz, respectively.

**Figure 6:**
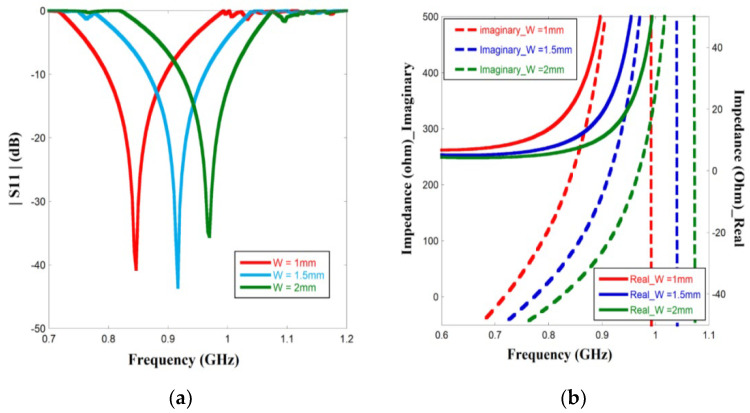
Parametric study of simulated (**a**) |*S*11| and (**b**) complex impedance for different W.

Next, varying the width of the radiating strip affects |*S*11|, and complex impedance was also evaluated. Figure 6a shows that as the width of the conducting strip increases, the resonant frequency also shifts towards a higher value. The wider radiating path increases the area, correspondingly restraining the tag to attain the required electrical length. Figure 6b shows the effect of width on the complex impedance, particularly the reactive part. Corresponding to the resonant frequency, the inductive value increased as the width increased.

### 3.2. Electric Field Vector Analysis of the Re-Configurable Antenna

**Figure 7:**
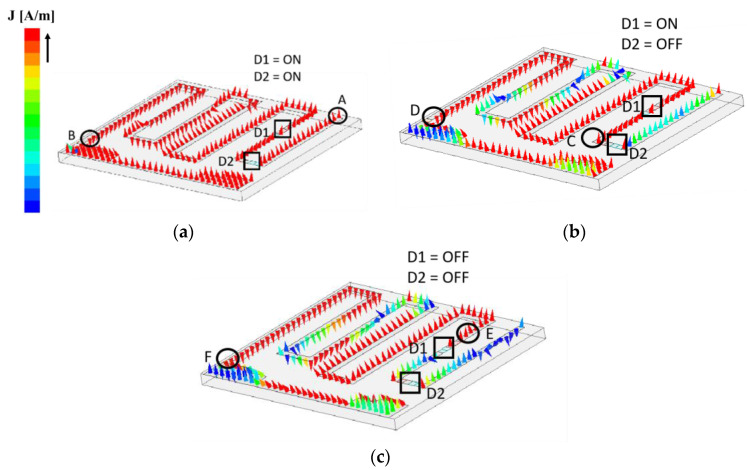
Electric field vector distribution for three different switching conditions: (**a**) 840 MHz; (**b**) 900 MHz; (**c**) 950 MHz.

The frequency switching mechanism of the proposed tag antenna is investigated through the simulated vector electric field distribution and is presented in Figure 7. Since the proposed antenna is designed with a pair of PIN diodes, four different conditions have been generated based on the ON/OFF status of the diodes. In this design, three conditions are considered, as shown in Figure 7a–c. For Figure 7a, both the diodes are in the ON condition, thereby providing a short-circuit path. Thus, as evident from Figure 7a, the entire length of the meandered radiator (A to B) is resonating and accommodating a full wavelength path corresponding to the resonant frequency of 840 MHz. As depicted in Figure 7b, the next condition is D1 is ON and D2 is OFF. Since the OFF condition indicates an open circuit, so, as highlighted in Figure 7b, the length of the meandered path given by C to D is resonating and accommodating a full wavelength path corresponding to the resonant frequency of 900 MHz while the path D to E is non-resonant, having a very insignificant amount of electric field strength. Following a similar phenomenon, both the diodes are OFF, and the path F to G accommodates the full wavelength path, thereby resonating at 950 MHz, as indicated in Figure 7c. 

### 3.3. Equivalent Circuit Analysis 

**Figure 8:**
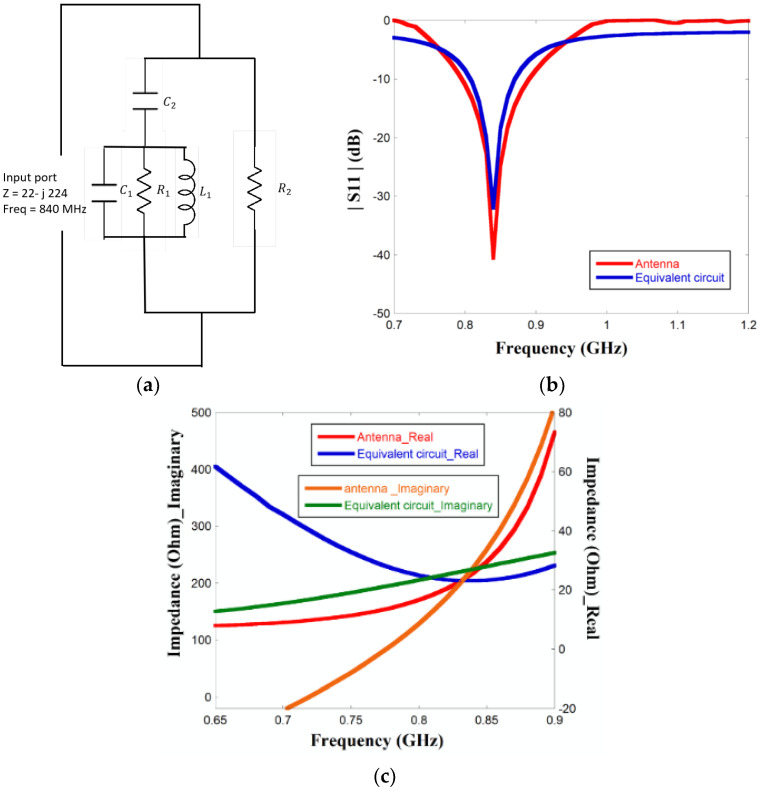
(**a**) Equivalent circuit of the proposed antenna. (**b**) Comparing results of |*S*11| between HFSS and ADS. (**c**) Comparing results of Impedance (Real and imaginary) between HFSS and ADS.

An equivalent circuit analysis of the proposed antenna is performed to highlight the impedance matching of the antenna with the chip. The equivalent circuit of the antenna is given in Figure 8a. Meander shape radiating strips can be considered short-end transmission lines, which are fundamentally composed of a parallel arrangement of radiating resistance (R1) and reactive components given by an inductor (L) and a capacitor (C1) [27]. A gap capacitance (C2) originates due to the gap between the ground and the meandered structure. The impedance (R2) indicates the intrinsic impedance of the substrate. $R_{2}$ can be calculated as [27]

(2) $$R_{2}=\sqrt{\frac{\mu_{0}}{\varepsilon }}\;$$

Here, $\mu_{0}$ is the permeability of the free space, which is equal to $1.25\times 10^{-6}\;H/m,$ and ε is the permittivity of the dielectric substrate FR4, where $\varepsilon =\varepsilon_{r}\;\varepsilon_{0}$. The relative permittivity of the FR4 substrate value is $\varepsilon_{r}$ = 4.4. The permittivity of free space is given by $\varepsilon_{0}=8.85\times 10^{-12}\;F/m$. The resonant frequency of the antenna can be calculated as [28],

(3) $$f_{r}=\frac{1}{2\pi \sqrt{L_{1}\left(C_{2}+C_{3}\right)}}\;$$

The equivalent circuit is modeled in Advanced Design System (ADS) software by considering the condition where both the diodes are in an ON state and the antenna is resonating at 840 MHz. From the simulation in ADS, the other circuit parameters are found to be satisfied. Figure 8b compares the *S*11 characteristic of the proposed antenna from full-wave simulation using HFSS and an equivalent circuit model using ADS. The figure indicates that both results are almost similar, thus confirming that the proposed equivalent circuit model is the correct interpretation of the antenna. From Figure 8c, the input impedance value of the antenna is 23 + j219 which is almost the complex conjugate of the chip impedance 22 − j224, thus indicating impedance matching required for maximum power transfer. Hence, no external impedance-matching network is required. 

Advanced Design System (ADS) software is used for the interpretation of the equivalent circuit. The value of the lumped components is unveiled from the software. The corresponding circuit is simulated in ADS software at 840 MHz without accounting for the PIN diodes. The main objective of the simulation is to signify the matching property of the recommended antenna without any external matching circuit. The comparable data from both the software HFSS and ADS of |*S*11| and antenna impedance are analyzed in Figure 8b,c. Figure 8a shows that return loss reached −40.6 dB and −32 dB at 836 MHz of the antenna and corresponding equivalent circuit, respectively. Therefore, the results validate that the meandering effect helps the antenna to resonate at the operating frequency and establish to match the complex input impedance.

## 4. Fabrication and Measurement 

**Figure 9:**
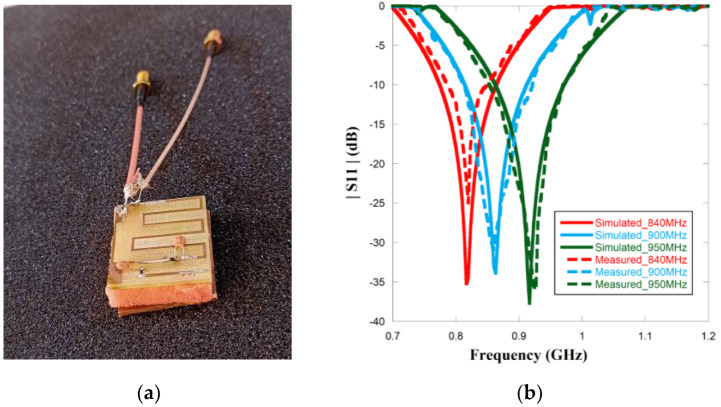
(**a**) Fabricated tag antenna with differential test fixture (**b**) |*S*11| results of simulated and measured data.

The photograph of the fabricated tag antenna with the metallic plate, PIN diodes, and UCODE microchip is presented in Figure 9a. The radiating plane is attached to the metal ground with the help of soft square foam material with a relative permittivity of 1.03, close to the air. The thickness of the foam is 5.4 mm, which is equal to the gap maintained between the tag and the metal plate. The photograph also depicts the measurement setup for the antenna. The |*S*11| parameter is measured with a vector network analyzer (VNA) of make Anritsu S820E. The VNA is calibrated over the frequency range of 800 MHz to 1 GHz. Generally, RFID antennae are balanced antennae and VNA has an unbalanced port with a 50 Ω coaxial connector. The differential method is one of the best methods for measuring the balanced antenna. In this method, a test fixture is created to connect the tag antenna to the VNA Port 1 and Port 2. Figure 9b shows the simulated and measured |*S*11| characteristic. The 3 V biasing is given to the corresponding PIN diodes to enable their ON and OFF conditions, and accordingly the frequency is also switched to three different points. The figure indicates that the measured results resemble the simulated results, and the switching characteristic is experimentally validated. 

**Figure 10:**
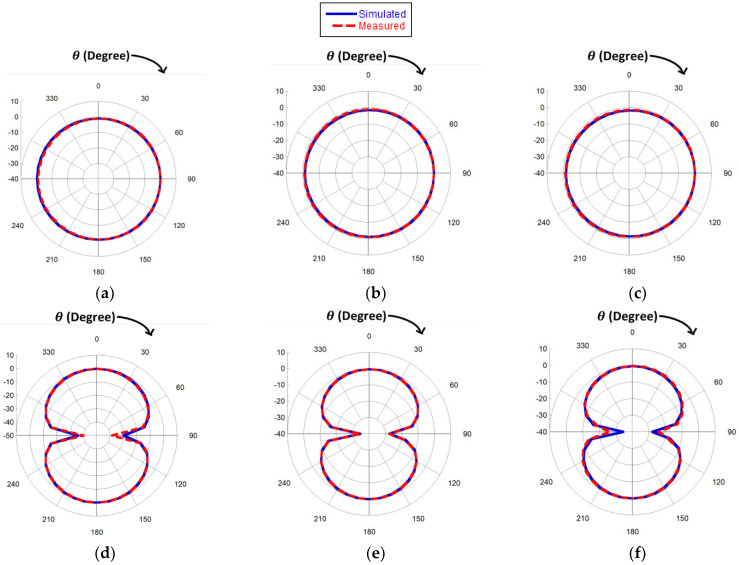
Normalized simulated and measured radiation pattern of XZ (**a**–**c**) and YZ plane (**d**–**f**) of the proposed antenna for respective frequencies 840 MHz, 900 MHz, and 950 MHz.

The radiation pattern is another key parameter to define the quality of the performance of the tag antenna. A tag can be attached to any surface at any angle. Therefore, the omnidirectional pattern helps the tag to detect an incoming signal from any direction. The simulated and measured radiation patterns of the proposed antennae are shown in Figure 10 for three switching frequencies. The figure highlights that the measured results satisfy the simulation results profoundly. A conventional omnidirectional pattern is obtained in XZ-plane as shown in Figure 10a–c and a bidirectional pattern in the YZ plane in both simulated and measured results as shown in Figure 10d–f, thereby indicating that the tag antenna should be positioned in the XZ plane.

Reading range is the crucial parameter to determine the distance from which a reader can detect the tag, which is of paramount importance in vehicle detection. The reading range can be calculated from the free-space Friis formula [19]:

(4) $$R=\frac{\lambda }{4\pi }\sqrt{\frac{P_{Tx}\;G_{R}\;G_{T}\;\tau }{P_{Th}}}$$

Here, λ is the wavelength of free space, $P_{Th}$ is the minimum threshold power to activate the microchip of the tag (tag sensitivity), $P_{Tx}$ is the transmitted power from the reader antenna, $G_{R}$ is the reader antenna gain, $G_{T}$ is the tag antenna gain, and τ is the transmission coefficient of the tag antennae. Equation (4) can be written as [19]

(5) $$R=\frac{\lambda }{4\pi }\sqrt{\frac{P_{EIRP}\;G_{r}\;}{P_{Th}}}$$

**Figure 11:**
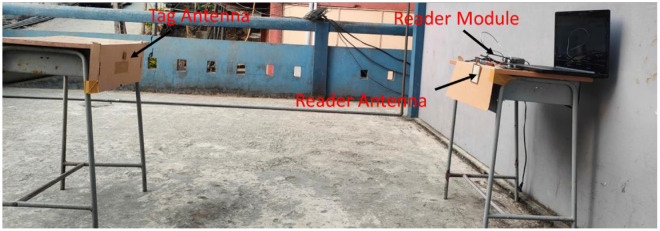
Measurement setup for read range in the free space for maximum distance.

**Figure 12:**
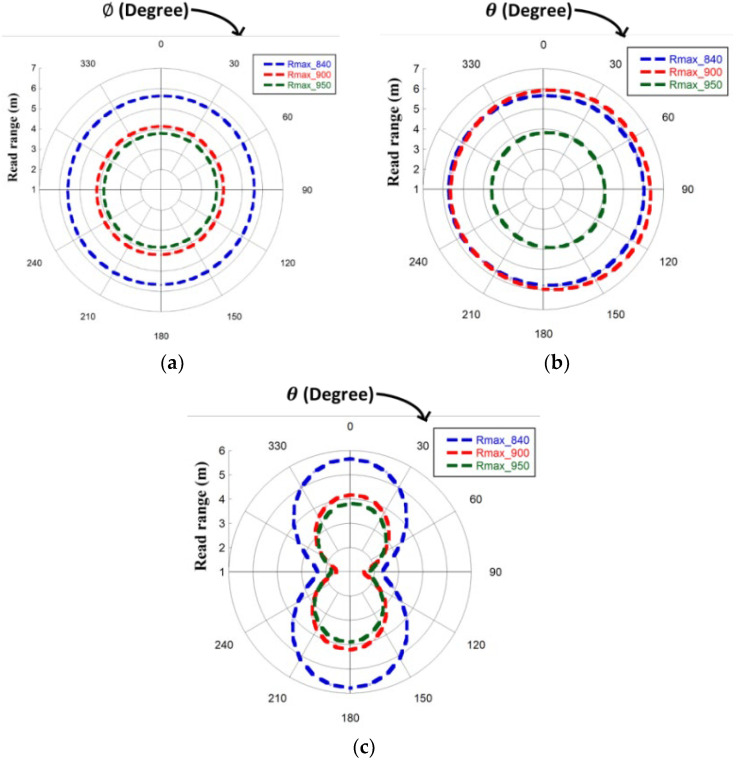
Measured Read range for three frequencies (840 MHz, 900 MHz, and 950 MHz) at (**a**) XY, (**b**) XZ, and (**c**) YZ planes.

$P_{EIRP}=$ $P_{Tx}.\;G_{R}\;$= permitted equivalent isotropic radiated power transmitted by the reader, and $G_{r}$ is the realized gain of the tag antenna and it is equivalent to $G_{T}\;.\;\tau$. The measurement setup for the read range determination is shown in Figure 11, which is carried out in an open space. The reader module (Identium Reader softV4.2) with a 3.32 dBi gain circular polarized Reader antenna is connected to the computer. Figure 12 shows the read patterns for three different frequencies for all three planes: XY, XZ, and YZ. Read distances of the tag antenna from the reader antenna are measured at the angular direction (θ,φ). The read patterns are generated by rotating the tag antenna over φ angles (keeping θ = 0°) for the XY plane, over θ angles (keeping φ = 0°) for the XZ plane, and over θ angles (keeping φ = 90°) for the YZ plane. A full omnidirectional read pattern is observed for all three switching frequencies in the XY plane with maximum read ranges of 5.67 m, 4.22 m, and 3.86 m at 840 MHz, 900 MHz, and 940 MHz, respectively. An almost omnidirectional read range pattern is observed in the XZ plane with a slightly increased read range at 90° for the switching frequencies. The maximum read range values at this plane are 6.04 m, 6.42 m, and 4.13 m at 840 MHz, 900 MHz, and 940 MH, respectively. A bidirectional read range pattern is noticed in the YZ plane for all three switching frequencies, with the maximum read range obtained at 0°. The values of the maximum read range are 5.78 m, 4.21 m, and 3.9 m at 840 MHz, 900 MHz, and 940 MH, respectively.

Table 4 illustrates the comparison of the performance of the proposed tag antenna with other reported works on UHF tag antennae. To the best of the knowledge of the authors, there is no literature proposing the switching action of RFID tag antennae. Therefore, the proposed work is compared with other metal-insensitive tag antennae in terms of size, realized gain, and read range. As compared to other structures [16,29,30,31,32,33], the proposed design has the lowest layout area, thereby proving to be more compact than others. The realized gain and read ranges are comparable to the other reported structures, even if the chip sensitivity of the proposed structure is less than most of the reported structures [29,31,32,33]. The read range is prominently better than [16,30,31,33]. Hence, considering the low sensitivity of the microchip used in the proposed structure, this design is the best candidate if a tradeoff is made among size, realized gain, and read range. The vital advantage of this structure is that it enables the switching function of the tag over triple frequencies without sacrificing the read range.

## 5. Conclusions

This paper has introduced and validated the switching operation of a metal mountable RFID tag antenna for three different frequencies at 840 MHz, 900 MHz, and 950 MHz, respectively. A high-power transmission coefficient (Near unity) has been achieved at all three switching frequencies. The biasing conditions of the two PIN diodes that have been incorporated in the tag antenna conveniently change the electrical length of the tag radiator, thereby causing the switching effect. In order to reduce the design complexity, the matching network has been avoided and the antenna port has been matched with the chip impedance by tuning the meander shape of the radiating element. To validate the impedance-matching results, the equivalent circuit of the proposed antenna has been validated in ADS software. Desired omnidirectional radiation pattern has been obtained on the H plane. The maximum read range is 6.42 m. The polar read range plot has been investigated in XY, XZ, and YZ planes and found to be satisfactory. The switching performances along with the read range confirm that the designed tag structure can be conveniently used in inter-country vehicle transportation. At all the switching frequencies, the realized gain is very low. Hence, future research may be directed towards the enhancement of gain of tag antennae using various passive engineered surfaces such as artificial magnetic conductors that will in turn enhance the read range.

## Figures and Tables

**Table 1:** Design Parameters of proposed antenna.

Parameter	Dimensions (mm)						
Substrate	L_S_		W_S_				
	30		34				
Radiating element	L_1_	L_2_	L_3_	L_4_	L_5_	L_6_	W
	24	20	23	4	13	4	1
Ground	L_G_	L_G1_		L_G2_			
	32.5	22		16			

**Table 2:** Comparison Results of Switching Conditions between Metal and Non-Metal.

Diode Condition		Resonant Frequency (GHz)		Impedance Bandwidth (%)	
D1	D2	Antenna without Metal Plate	Antenna with Metal Plate	Antenna without Metal Plate	Antenna with Metal Plate
ON	ON	0.835	0.82	11	10.9
ON	OFF	0.88	0.86	11.8	12.7
OFF	OFF	0.93	0.92	11.9	10.8

**Table 3:** Impedance Value of Different Frequencies.

Average Impedance of Microchip is 22 − j224 Ω	**Frequency (MHz)**	**Impedance (Ω)**
	835	28 + j218
	880	24 + j219
	930	28 + j223

**Table 4:** Comparison of different metal mountable Tag Antennae.

Ref	Tag Size	Resonant Frequency (MHz)	Input Impedance (Ohm)	Power (W)	Chip Sensitivity (dBm)	Realized Gain (dB)	Max Read Range (m)
[16]	0.26 $\lambda_{0}$× 0.26$\;\lambda_{0}$	917	6.17 − j162.97	4	−17.8	−5.78	6.5
[29]	0.12 $\lambda_{0}$× 0.122 $\lambda_{0}$	915	12.1 − j161.51	4	−20	−5.08	7
[30]	0.116 $\lambda_{0}$× 0.116 $\lambda_{0}$	915	14.6 − j161.25	4	−17.8	−6.84	5.65
[31]	0.12 $\lambda_{0}$× 0.152 $\lambda_{0}$	912	11.9 − j118.94	4	−20	−8	5.2
[32]	0.12 $\lambda_{0}$× 0.152 $\lambda_{0}$	915	20.9 − j193.16	4	−20.5	−4.11	8.14
[33]	0.156 $\lambda_{0}$× 0.156 $\lambda_{0}$	867	13.4 − j126.1	3.28	−20	−8	5.52
This work	0.083 $\lambda_{0}$× 0.094 $\lambda_{0}$	836 889 930	22 − j224	4	−18	−8.4 −9.2 −9.5	6.04 6.42 4.21

## Data Availability

Data are available as needed from the corresponding author.
